# Evaluation of Knowledge, Attitudes, and Practices about Exclusive Breastfeeding among Women in Italy

**DOI:** 10.3390/ijerph16122118

**Published:** 2019-06-14

**Authors:** Diana Cascone, Davide Tomassoni, Francesco Napolitano, Gabriella Di Giuseppe

**Affiliations:** Department of Experimental Medicine, University of Campania “Luigi Vanvitelli”, Via Luciano Armanni, 5 80138 Naples, Italy; dianacascone82@gmail.com (D.C.); davidetomassoni@hotmail.it (D.T.); francesco.napolitano2@unicampania.it (F.N.)

**Keywords:** exclusive breastfeeding, knowledge, Italy, practices, survey, women

## Abstract

Background: The aim of this study was to assess the level of knowledge, attitudes and behaviors of women about breastfeeding in Italy. Methods: A cross-sectional survey was carried out between January and June 2016 in the Campania Region among mothers who were going to six public vaccination centers. Data were collected by two researchers through face to face interviews. Results: Two thirds of the women had heard on exclusive breastfeeding (64.6%) and the 71% of them knew that exclusive breastfeeding should be practiced for at least six months. Nearly all mothers had breastfed their child (93.2%), but only 33.3% of them had practiced exclusive breastfeeding for at least six months. Women who agree that breastfeeding creates a positive relationship between the mother and the child, who practiced exclusive breastfeeding during the hospital stay, and who had received breastfeeding advice at hospital discharge were more likely to practice exclusive breastfeeding for at least six months. Conclusions: The results of this survey may be helpful to policy makers and managers when planning educational interventions on breastfeeding both during pregnancy and during hospital admissions for delivery. Indeed, there is a need to increase efforts to make mothers aware of health benefits of breastfeeding for themselves and their offspring during their hospital stay after delivery. This research has the potential to increase exclusive breastfeeding rates and subsequent maternal and child health outcomes.

## 1. Introduction

Poor early childhood nutrition can negatively impact a child’s physical and emotional development in both the short and long-term, and limit adult achievement and productivity. It is well known that breastfeeding is recommended to provide young infants with the nutrients they require for healthy growth and development [1]. In fact, it has been widely demonstrated that breastfeeding reduces the risk of certain infections and non-communicable diseases [2,3,4], and for mothers, reduces the risk of breast and ovarian cancer [5,6]. In addition, breastfeeding provides economic and environmental advantages to society [7].

The World Health Organization and the American Academy of Pediatrics recommend exclusive breastfeeding for up to six months. This should be followed by continued breastfeeding as complementary foods are introduced, and a continuation of breastfeeding until the child reaches two years of age or beyond, as mutually desired by the mother and the infant [8,9]. 

It is well known that women’s socio-demographic characteristics, will, and level of motivation are essential in determining their decision to comply with appropriate breastfeeding practices [10,11]. However, pediatricians, midwives, and general practitioners also play an important role in the promotion of breastfeeding.

Several global studies were conducted that evaluated primarily the attitudes and behavior of mothers in relation to exclusive breastfeeding. Most of these studies were conducted in developing countries [12,13,14,15,16,17,18,19,20,21], and only few were run in industrialized countries [22,23,24,25,26,27]. Therefore, the present survey aims to investigate the knowledge, attitudes, and practices of women towards exclusive breastfeeding to determine their reasons for why breastfeeding was interrupted or not carried out, and analyze the factors associated with knowledge and practices surrounding exclusive breastfeeding in the six first months of their child’s life.

## 2. Materials and Methods

### 2.1. Setting

A cross-sectional study was carried out between January and June 2016 in the Campania Region among mothers taking their children to one of six public vaccination centers. The vaccination centers were selected for the recruitment procedure because in Italy mandatory vaccination rates have reached values >93% in children aged one to two years [28]. Therefore, the selected mothers can be considered a representative sample.

### 2.2. Participants

The sample was selected using a two-stage sampling method. In the first stage, from the list of 147 regional vaccination centers, six centers were selected through a simple random sampling (primary sampling units). In the second stage, mothers of children less than two years-old who visited the vaccination center on recruitment days were randomly selected and invited to undergo an interview (secondary sampling unit). Mothers were eligible if they were aged 18 years or over, had children without congenital diseases, and were without acute or chronic respiratory diseases. In addition, mothers were excluded if they self-reported to have a chronic infectious disease (such as acquired immune deficiency syndrome or hepatitis B) or if they were mothers of twins or adoptive children.

### 2.3. Sample Size

The sample size was determined assuming that 34% of mothers practice exclusive breastfeeding for up to six months as in accordance with the average of the previous values reported in published literature [23,24,25,29,30]; a 95% confidence level, a 5% margin of error and a 70% response rate. Therefore, in order to obtain a representative sample of the population, the final sample size was calculated to comprise of 450 women.

### 2.4. Data Collection

Before beginning the study, the vaccination centers’ directors received a letter requesting collaboration and approval, and explaining the purpose of the investigation and the methods of data collection.

Data were collected through face-to-face interviews with the women on a randomly chosen working weekday during the study period. Two researchers approached the mothers who visited the vaccination centers and those who gave consent were interviewed until the final sample number was reached. The interviews were performed in the waiting rooms of the centers using a questionnaire. The interviews lasted 15 minutes on average and were performed to be as private as possible. This was done in order to maximize the chances of the participants feeling comfortable and able to answer the questionnaire. Before beginning the interview, the researchers described the survey topic and the organization of the questionnaire to the participants and assured them that the interview was completely anonymous and that the data collected would be kept private, and that the written informed consent was collected. In addition, the research team explained to the women that they could stop the interview at any time without penalty, that the participation was voluntary, and that no payment would be given.

### 2.5. Instrument

The items included in the questionnaire were chosen based on previous published investigations [12,15,25,29,31,32] or because they were considered interesting, or to address the aims of the study by the research team. The questionnaire consisted of the following six sections: (1) The demographic characteristics of the women (age, level of education, marital and employment status, number of pregnancies, number of children and number of adults cohabiting); (2) the mother’s medical history regarding pregnancy, childbirth and children (pre-pregnancy health problems such as having at least one chronic condition, pregnancy health problems such as pre-eclampsia, placenta previa, gestational diabetes, and threat of preterm labor place of birth complications during childbirth, number of hospital days after childbirth child’s date of birth child’s gender pregnancy week in which the child was born, child’s birth weight and whether they practiced rooming in (keeping mother and infant together in the same room after birth) during hospitalization); (3) the mother’s knowledge about breastfeeding, in which response options for questions concerning breastfeeding included “yes”, “no” or “do not know”; (4) the mother’s attitudes on the benefits and limitations of breastfeeding, in which: Attitudes were measured with questions that required a “agree”, “uncertain” or “disagree” responses or a 10-point Likert scale response that ranged from 1 to 10 [33]; (5) the mother’s behaviors regarding breastfeeding and child nutrition and any reasons given by mothers stopping breastfeeding, in which breastfeeding practices were measured by questions that required a nominal or categorical (yes or no) or open format responses, and (6) the mother’s primary source of information and their need for any of additional information on breastfeeding.

For the evaluation of women’s knowledge and practices we considered exclusively breastfeeding to mean feeding an infant with only breast milk with the exception oral rehydration solution, and drops/syrups of vitamins, minerals or medicines in accordance with the definition provided by the World Health Organization [8].

Prior to conducting this investigation, a pilot study was carried out among a sample of 25 women (not included in the final sample) to evaluate the comprehensibility and the validity of the questions. Following this, no changes were made to the questionnaire.

### 2.6. Ethics

The study protocol was approved by the Ethics Committee of the Teaching Hospital of the University of Campania “Luigi Vanvitelli”. The ethical approval code number is: 922/6.26.2015.

### 2.7. Statistical Analysis

Following a descriptive analysis of the data, the inferential analysis was conducted according to a double stage strategy. In the first stage, two multivariate logistic regression full models were built to evaluate the effect of each independent variable on the following outcomes of interest: (1) Women who have heard about exclusive breastfeeding and know that it should be practiced for at least six months (no = 0; yes = 1) (Model 1); (2) profile of mothers who practiced the exclusive breastfeeding for up to six months (no = 0; yes = 1) (Model 2). The selection of variables included in the models was done considering previous investigation in published literature, while other variables were chosen because they were considered as interesting predictors of the outcomes. 

In the second stage, the variables included in the final models were determined using a stepwise backwards removal method deleting variables with a p value above 0.4 in order to exclude not so important variables from the model until the minimum adequate model was reached and the Hosmer and Lemeshow test was used to assess the goodness-of-fit of the final models [34]. 

In Model 1 and Model 2, the following independent variables were included: Age (continuous, in years), nationality (others = 0; Italian = 1), marital status (unmarried = 0; married = 1), educational level (three categories: No formal education, elementary or middle school = 1; high school = 2; college degree or higher = 3), occupation (unemployed = 0; employed = 1), week of pregnancy in which the child was born (<37 weeks = 0; ≥37 weeks = 1), weight of the child at birth (<2.5 kilograms = 0; ≥2.5 kilograms = 1), type of hospital in which the delivery occurred (private = 0; public = 1), type of delivery (cesarean = 0; vaginal = 1), rooming-in for infants (no = 0; yes = 1), sources of information about breastfeeding (others = 0; healthcare providers = 1), health problems related to pregnancy (no = 0; yes = 1), and needs of additional information about breastfeeding (no = 0; yes = 1). 

In Model 2, the following variables were also included: Adequate knowledge regarding the exclusive breastfeeding (no = 0; yes = 1), positive attitude about the benefits of exclusive breastfeeding for the child health (ordinal variable), positive attitude about the benefits of exclusive breastfeeding for the mother’s health (ordinal variable), mother who had previously breastfed other children (no = 0; yes = 1), type of nutrition for the infant recommended to hospital discharge (other types of feeding = 0; exclusive breastfeeding = 1), having fed the infants in the hospital with exclusive breastfeeding (no = 0; yes = 1), agree that breastfeeding is the best nutrition for the infant in the first six months of life (no = 0; yes = 1), agree that breastfeeding is the simplest nutrition for the mother (no = 0; yes = 1), agree that breastfeeding creates a positive relationship between mother and child (no = 0; yes = 1).

In the logistic regression models, odds ratios (ORs) as well as their 95% confidence intervals (CIs) were calculated performing the analyses. All statistical tests were two-tailed and differences were considered to be statistically significant at a *p*-value less than or equal to 0.05. All analyses were performed using Stata version 10.1 statistical software (StataCorp LLC: College Station, TX, USA) [35].

## 3. Results

### 3.1. Women’s Characteristics

A total of 506 out of the 523 women recruited gave consent to be interviewed, which yielded a response rate of 96.7%. The main characteristics of the sample are described in Table 1. In particular, the average age was 31.5, just over half of the women (54.1%) had had more than one pregnancy and 44.5% had more than one child. In addition, nearly all had given birth at full term (94.3%) and three-quarters of the women had practiced rooming-in.

### 3.2. Breastfeeding Knowledge 

The results concerning the knowledge of the study participants are described in Table 2. In total, two thirds of the women had heard about exclusive breastfeeding (64.6%), and 71% of them knew that exclusive breastfeeding should be practiced for at least six months. Only 48% of women with one child had this knowledge. Nearly all (92.9%) of the participants knew that breast milk contains antibodies that are transferred to the baby and 84.8% knew that breast milk reduces the risk of certain infectious diseases. In addition, 57.5% of women correctly answered that breastfeeding reduces the risk of some non-communicable diseases (asthma, obesity and diabetes), while only 45.5% and 31% correctly indicated that it reduces the risk of breast cancer and of some non-communicable diseases (diabetes, obesity and osteoporosis) respectively for mothers.

The results of the logistic regression model showed that women who were older (OR = 1.06: 95% CI 1.01–1.11), married (OR = 3.3; 95% CI 1.64–6.64), had a college degree or higher education (OR = 3.38; 95% CI 1.47–7.81) as compared to women with no formal education, elementary or middle school, those who had practiced rooming in (OR = 2.2; 95% CI 1.39–3.49), and those who had healthcare providers as their source of information on breastfeeding (OR = 2.67; 95% CI 1.48–4.83) were more likely to have heard about the exclusive breastfeeding and to know that it should be practiced for at least six months (Model 1 in Table 3).

### 3.3. Attitudes Towards Exclusive Breastfeeding

The participants believed that the exclusive breastfeeding is very important for both the child’s and the mother’s health with average values of 9.6 and 8.7, respectively, out of a maximum score of 10. The majority believed that breastfeeding provides the best nutrition for the infant in the first six months of life (91.1%), believed that breastfeeding is the simplest nutrition for the mother (61.9%), and agreed that it creates a positive relationship between mother and child (76.3%). Moreover, only 28.8% of the mothers agreed that exclusively breastfeeding helps in losing weight gained during pregnancy, while 39.2% agreed that it delays the mother’s return to work.

### 3.4. Breastfeeding Practices 

Some questions were intended to investigate how newborns were fed during their hospitalization. According to the responses received, 59.7% of women reported that their child received only breast milk, 17.6% received breast milk and formula milk and 6.2% received only formula milk. During their hospital discharge, more than two thirds of the women (68.2%) received advice on exclusive breastfeeding, while 21.5% received advice on both breast and formula milk and only 7.1% received advice on formula milk. Nearly all the mothers had breastfed their last child (93.2%) and 87.1% of women with more than one child had breastfed previously. Only 33.3% of mothers practiced exclusive breastfeeding for at least six months. Women who agree that breastfeeding creates a positive relationship between mother and child (OR = 1.89; 95% CI 1.07–3.33), those who practiced exclusive breastfeeding during their hospital stay (OR = 3.34; 95% CI 1.99–5.62), and those who received a recommendation to breastfeed exclusively during their hospital discharge (OR = 2.54; 95% CI 1.41–4.58) were more likely to practice exclusive breastfeeding for at least six months (Model 2 in Table 3).

Furthermore, 40.9% of women weaned their child before they were six months old, but a third (34.5%) breastfed their child even after six months. The women interviewed could indicate one or more reasons why they had stopped breastfeeding and the most frequently reported reasons were, in order as follows: their maternal perception of insufficient breast milk (65.5%), difficulty and pain during breastfeeding (19.5%), voluntary termination because it was stressful (17.6%), and an inadequate increase in the weight of the child (5.7%).

### 3.5. Sources of Knowledge

The participants indicated that they mainly acquired their breastfeeding knowledge from physicians (51.6%) and friends (42.2%). Other sources included, in order, prenatal education courses (13.2%), newspapers/broadcast television (10.6%), and the internet (6.1%). In addition, 76.1% of mothers considered the level of information received from physicians as good (58.7%) or excellent (17.4%). Finally, approximately one-third (31.2%) of the women reported the need to receive additional information on breastfeeding.

## 4. Discussion

The results of this study expanded the few previous reports conducted in developed countries relating to mothers’ knowledge, attitudes, and behaviors concerning breastfeeding. One of the study’s main results is that most women have adequate knowledge on exclusive breastfeeding and its relative benefits for the child and the mothers. Indeed, nearly all of the participants knew that breast milk reduces the baby’s risk of infectious diseases, and approximately two thirds knew that breastfeeding reduces the risk of some non-communicable diseases (asthma, obesity and diabetes).

A comparison of the results of this study with those of other investigations on the same topic is rather difficult due to the different aims, study methodologies, study samples, and geographical areas of interest. However, our results concerning the levels of knowledge on exclusive breastfeeding and its benefits have been confirmed in previous studies conducted in Ireland [36], Egypt [12], Australia [37], Kenya [16], Ghana [17], and Ethiopia [20].

The results of this survey showed that almost all mothers have breastfed their children, but only 33.3% practiced exclusive breastfeeding even though most women have heard of exclusive breastfeeding and consider it important for the health of the baby and the mother. This value indicates that healthcare providers who care for mothers should increase their efforts to promote breastfeeding and that there is a need for public policies which that ensure the living and working conditions of women are compatible with breastfeeding.

The study’s findings regarding exclusive breastfeeding are similar to those reported by previous investigations conducted in Canada, Egypt, and Nigeria where, respectively, 26%, 32% and 33.5% of mothers breastfed their infants for at least six months [12,13,30]. The proportion of mothers who breastfeed their children was higher in a survey conducted in Guatemala, Honduras, Mexico, Nicaragua, Panama, and El Salvador where the number of women who practiced exclusive breastfeeding ranged from 44.5% to 76.8% [38], among women in Ethiopia (68.6%) [39], and among women in Ghana (58%) [17]. The lowest percentages of women who practiced exclusive breastfeeding were reported in Norway [40], China [41], and in two studies conducted in United States [29,42].

The more frequently reported reasons for why mothers stopped breastfeeding were maternal perceptions of insufficient breast milk, difficulty and pain during breastfeeding, voluntary termination because it was stressful, and an inadequate increase in the weight of the children. Insufficient breast milk and poor infant growth for their age have been reported as reasons for stopping breastfeeding in already cited surveys [39]. In addition, an insufficient breast milk supply, the inadequate weight of children, and pain and stress associated with breastfeeding were reported in a survey of Western Australian mothers [31]. Finally, maternal perception of having an inadequate breast milk supply was a frequently reported reason for the discontinuation of the breastfeeding among women in Ireland [32], United States [43], and Australia [44].

According to a multivariate analysis, practicing exclusive breastfeeding during the hospital stay and receiving a recommendation to breastfeed during the hospital discharge were the factors most strongly associated with the practice of exclusive breastfeeding. These results highlight the key role of healthcare providers in increasing appropriate behaviors regarding breastfeeding and, therefore, the importance of women receiving proper information. Other previous studies have also confirmed the key role and influence of correct information on breastfeeding provided by healthcare professionals [39,45,46]. In addition, women with a college degree or higher level of education were more likely to have heard about exclusive breastfeeding and to know that it should be practiced for up to six months. This result is consistent with other previous studies conducted among women in the same geographical area, which confirmed the positive impact of a high level of educational on the subject’s knowledge related to the health topics [47,48,49,50,51].

The present survey has some limitations that must be considered in order to properly interpret the results. The first limitation involved the design of the cross-sectional study which was unable to determine a causal relationship between outcomes of interest and independent factors. Second, behaviors concerning the duration of breastfeeding, reasons for stopping, and any advice given or not given during their hospital stay were measured by self-reported data provided by the interviewed women. Therefore, it is possible that recall bias occurred which would lead to an inaccurate evaluation of the outcomes of interest. However, although it is not possible to exclude that a recall bias has occurred, a salient event, such as breastfeeding, is less likely to be forgotten. Furthermore, mothers were asked to be very accurate in their recall of the details concerning breastfeeding. Third, the methodology of data collection through face-to-face interviews, instead of the self-administration of questionnaires, could result in overestimation of the practices related to exclusive breastfeeding because the respondents may have reported more socially desirable behaviors. However, personal interviews were chosen for the advantage of achieving a high response rate, a level of accuracy in selecting respondents and the avoidance of missing data. Finally, this study considered a sample of mothers who visited six public vaccination centers in the Campania region which could lead to different results than those of analogous samples in the other Italian regions. Therefore, although this study cannot eliminate the idea that the results of this study pertain only this area, it is reasonable to assume that the main characteristics of the mothers of less than two-year-old children participating in this study provide a sample to other regions of Italy. On the other hand, this study has certain strengths. In particular, the sample size is large and was carefully selected and the response rate is very high. In addition, this study assessed not only the knowledge and behaviors related to breastfeeding, but also the factors associated with exclusive breastfeeding knowledge and practices. Finally, because a limited amount of data is available on the knowledge and behaviors of mothers concerning breastfeeding in Italy and other developed countries, this study helps to increase knowledge on this issue.

## 5. Conclusions

The results of this study highlight the need for policy makers and healthcare providers to direct their efforts to providing evidence-based information and recommendations to mothers on the benefits of breastfeeding. Indeed, the findings of this study are showed that recommendation on exclusive breastfeeding given during a mother’s hospital discharge and the practice of breastfeeding during the mother’s hospital stay have a strong correlation with the continued practice of exclusive breastfeeding among mothers. These results may be helpful to policy makers and managers as they plan educational interventions on breastfeeding during both pregnancy and hospital admissions during delivery. In addition, healthcare providers should support mothers by offering educational interventions and help them to overcome difficulties they have while breastfeeding. Mothers may experience stress and pain in the early stages of breastfeeding and have misconceptions about the appropriate amount of milk necessary for proper child growth. Implementing these efforts is crucial to increasing rates of exclusive breastfeeding for the first six months of a child’s life and the subsequent improvement in both the mother’s and the child’s health outcomes.

## Figures and Tables

**Table 1:** Socio-demographic and maternal characteristics.

Characteristics	*n*	%
Age (years)	31.5 ± 6.1 (18–48)	
<30	194	38.3
≥30	312	61.7
Nationality		
Italian	435	86
Other	71	14
Educational level		
College degree or higher	148	29.3
High school	247	48.8
No formal education, elementary/middle school	111	21.9
Marital status		
Married	417	82.4
Other	89	17.6
Employment status		
Employed	235	46.4
Unemployed/Housewife	271	53.6
Number of pregnancies	1.75 ± 0.85 (1–6)	
1	232	45.9
2	191	37.7
≥3	83	16.4
Number of children	1.53 ± 0.66 (1–4)	
1	281	55.5
≥2	225	44.5
Pre-pregnancy health problems		
Yes	75	14.8
No	431	85.2
Pregnancy health problems		
Yes	67	13.2
No	439	86.8
Place of birth		
Public hospital	330	65.2
Private hospital	176	34.8
Type of birth		
Vaginal delivery	279	55.1
Caesarean section	227	44.9
Gestational age	39.3 ± 1.6 (27–42)	
Premature (<37 week)	29	5.7
Full Term (37–42 week)	477	94.3
Child’s body weight at birth	3.2 ± 0.4 (1.4–4.6)	
<2.5 Kgs	17	3.4
≥2.5 Kgs	489	96.6
Rooming-in		
Yes	379	74.9
No	127	25.1

**Table 2:** Knowledge about breastfeeding of the study population.

**Knowledge**	**Yes**		**No**			
	***n***	**%**	***n***	**%**		
Have heard about the exclusive breastfeeding	327	64.6	179	35.4		
Knowledge that exclusive breastfeeding should be practiced at least six months ^a^	232	71	95	39		
	**Correct Response**		**Incorrect Response**		**Do Not Know**	
	***n***	**%**	***n***	**%**	***n***	**%**
Breast milk contains antibodies that are transferred to the baby	470	92.9	5	0.9	31	6.2
Breast milk may protect the baby against infectious diseases	429	84.8	29	5.7	48	9.5
Breastfeeding protects the baby against some chronic conditions (asthma, obesity, diabetes)	291	57.5	78	15.4	137	27.1
Breastfeeding protects the women by the breast cancer	230	45.5	104	20.5	172	34
Breastfeeding protects the women by the onset of certain chronic diseases (diabetes, obesity, osteoporosis)	157	31	127	25.1	222	43.9
Breastfeeding should be avoided in case of cold or flu mother	369	72.9	47	9.3	90	17.8

^a^ Only for those who reported that they have heard about exclusive breastfeeding.

**Table 3:** Multivariate logistic regression analyses indicating associations between independent variables and the different outcomes.

Variable	OR	SE	95% CI	*p*-value
Model 1. Having heard about the exclusive breastfeeding and knowledge that should be practiced at least 6 months				
Log likelihood = −154.63, χ^2^ = 79.85 (10 df), *p* < 0.0001				
Marital status	3. 3	1.17	1.64–6.64	0.001
Mothers who have practiced the rooming in	2.2	0.52	1.39–3.49	0.001
Healthcare providers as source of information about breastfeeding	2.67	0.81	1.48–4.83	0.001
Older age	1.06	0.02	1.01–1.11	0.028
Educational level				
No formal education, elementary or middle school	1 *			
High school	1.45	0.55	0.69–3.06	0.33
College degree or higher	3.38	1.44	1.47–7.81	0.004
Children’s body weight at birth ≥2.5 kilograms	7.71	8.55	0.87–67.91	0.07
Type of delivery	1.66	0.49	0.94–2.96	0.082
Needs of additional information about breastfeeding	0.69	0.21	0.39–1.25	0.222
Health problems related to pregnancy	1.44	0.58	0.65–3.15	0.365
Model 2. Profile of mothers who practiced the exclusive breastfeeding for at least six months				
Log likelihood = −269.82, χ^2^ = 87.11 (8 df), *p* < 0.0001				
Women who practiced exclusive breastfeeding during the hospital stay	3.34	0.88	1.99–5.62	<0.001
Women who had received the exclusive breastfeeding recommendation at hospital discharge	2.54	0.76	1.41–4.58	0.002
Agree that breastfeeding creates a positive relationship between mother and child	1.89	0.55	1.07–3.33	0.03
Marital status	1.77	0.52	0.99–3.16	0.051
Type of hospital in which the delivery occurred	1.57	0.37	0.99–2.51	0.055
Occupation	1.38	0.3	0.9–2.12	0.132
Week of pregnancy in which the child was born	1.39	1.15	0.73–6.14	0.164
Agree that the exclusive breastfeeding is very useful for the health of child	1.54	0.53	0.78–3.03	0.210

* Reference category. OR: odds ratio; SE: standard error; CI: confidence interval.
